# Quantitative Assessment of Apically Extruded Debris after Single-Files Supplemental Retreatment considering Apical Patency Influence: *In Vitro Study*

**DOI:** 10.1155/2022/7544813

**Published:** 2022-12-21

**Authors:** Neveen Ali Shaheen, Nahla Gamaleldin Elhelbawy, Dalia Abdelhamid Sherif

**Affiliations:** ^1^Endodontic Department, Faculty of Dentistry, Tanta University, Tanta, Egypt; ^2^Dental Biomaterials Department, Faculty of Dentistry, Tanta University, Tanta, Egypt

## Abstract

**Objective:**

This study aims to assess the impact of re-establishing apical patency on the quantity of debris extruded through the apex after three supplementary retreatment files (TruNatomy (TRN), WaveOne Gold (WOG), and XP endo Finisher R (XPFR)).

**Materials and Methods:**

Eighty single-rooted mandibular premolars were prepared with ProTaper Universal rotary systems (PTU) up to F3 and obturated. The samples were divided into two main groups according to the presence or absence of the apical patency (*n* = 40), GI with apical patency and GII without apical patency. Based on the file system, which was adopted to eliminate the previous filling, each group had four subgroups (*n* = 10). In GI PTUR and GII PTUR, ProTaper Universal retreatment files (PTUR) were utilized only to remove the most primary filling material (control groups). In the other groups (PTUR) used, it was first followed by supplementary files. The quantity of debris extruded by different retreatment file systems was determined and compared to the corresponding control group with or without apical patency. Data were analyzed using a two-way ANOVA with a post hoc Tukey's multiple comparison test at a 5% significance level.

**Results:**

There was a statistically significant difference among the control and experimental groups. XPFR had the least quantity of extruded debris. Apical patency did not affect the debris extrusion.

**Conclusion:**

All tested files led to a significant apical debris extrusion regardless of apical patency.

## 1. Introduction

Nonsurgical endodontic retreatment is the first plan for failed endodontic treatment. It entails removing the root canal filling material to gain access to the apical area, followed by cleaning, shaping, and refilling [1]. Different removal methods revealed remnant filling materials at various canal levels; therefore, cleaning the apical foramen appears to be crucial during retreatment procedures. On the contrary, overinstrumentation during the retreatment may force debris and filling materials into the periapical area or even induce transportation of the apical portion of the canal [2, 3]. Debris extrusion is one of the difficulties encountered during endodontic treatment and is accompanied by a high rate of postoperative pain, flare-ups, and possibly apical healing failure [4–6]. Regardless of the method, apical debris extrusion occurs during obturating the material removal. It remains in the apical third and its quantity is governed by the instrument design and motion kinematics [7–9].

Appropriate cleansing of the apical foramen and re-establishing of apical patency is regarded to be critical for a favorable outcome following nonsurgical endodontic retreatment [10]. The American Association of Endodontists defines patency as “a canal preparation technique in which the apical region is kept debris-free via the recapitulation with a small file through the apical foramen” [11]. Buchanan defines patency as a “small flexible K-file passively advanced 0.5–1 mm beyond the minor diameter without enlarging it” [12]. So, the patency maneuver entails entering the root canal using an instrument compatible in size with the actual length of the tooth to clear the whole root canal of debris during the instrumentation [13, 14].

Previous studies have reported contradictory findings regarding the necessity of apical patency during the endodontic treatment, with some research indicating that it is not always necessary [15, 16]. Others believe that cleaning the apical foramen following apical patency was a significant predictor of endodontic performance in general and endodontic retreatment in particular [10] as apical patency had been related to a lower incidence of postoperative pain [17]. On the contrary, multiple investigations have revealed a link between apical patency and the extrusion of contaminated debris and irritated periapical tissues especially when using manual preparation approaches [18–21].

Hand-operated, rotary, or ultrasonic equipment, either with or without using solvents, have been used to remove root canal fillings. However, the total removal of the obturating filling is not yet attainable with the presently available retreatment processes. Consequently, supplemental approaches to increase the removal of residual filling material and clean root canals have been proposed [22]. Rotary nickel-titanium (NiTi) devices are recommended for endodontic retreatment procedures due to their safety, efficiency, and rapidity, in addition to the friction generated that plasticizes the gutta-percha, allowing its removal [23]. The development of single-file rotary (NiTi) systems that reduces the number of files used may minimize apical debris extrusion and symptomatic apical periodontitis [24].

PTUR files are composed of three instruments: D1 (30/0.09), D2 (25/0.08), and D3 (20/0.07). WaveOne Gold (WOG) is a single-file system with parallelogram cross-section, changeable tapers, and a semiactive tip, and it comes in four sizes [25]. Debris extrusion by this file system shows contradictory results [26–28].

The XP-endo Finisher R instrument (XPFR) is a single-file system with a continuous rotation motion designed for retreatment. Due to its bigger core diameter of size 30 and zero-taper, it is stiffer and consequently more aggressive and efficient in displacing root filling materials left over from traditional procedures [22, 29, 30]. To the authors knowledge, there is a paucity of data on the apically extruded debris by this file system during retreatment [31, 32].

TruNatomy instruments (TRN) are innovative, heat-treated NiTi instruments available in three sizes. They have a varying regressive taper, an off-center parallelogram square cross-section and has been heat-treated to improve its elasticity and resistance to cyclic fatigue [33, 34]. There is a scarcity of data on assessing extruded debris apically during the root canal filling material elimination using the TRN system as a supplementary approach [35–37].

During the retreatment, there were contradictory results about the importance of apical patency and the link between it and the quantity of apical extrusion of debris. In addition to the insufficient and contradictory information about how much debris is expelled by three contemporary single-file systems (WOG, TRN, and XPFR) used to remove root canal filling during the retreatment. Therefore, this study's purpose was to assess the quantity of debris extruded apically after using a twofold approach for the removal of previous root filling materials with a supplementary single file system after using PTUR files either with re-establishing apical patency or without it. The study hypothesized a correlation between apical patency and apical debris extrusion. Furthermore, no variation of apically extruded debris will be found among the three supplementary approaches.

## 2. Materials and Methods

This study was approved by the Research Ethics Committee of Tanta University Faculty of Dentistry, Egypt, in March 2022. Eighty extracted single-rooted mature premolars with a single root canal and a degree of curvature less than 20° were used [38]. Periodontal disease or orthodontic treatment necessitated the extraction of these teeth. The teeth with significant caries, root resorption, calcification, an open apex, prior endodontic treatment, or root fracture were excluded from the study. The teeth were soaked in 5.25% sodium hypochlorite for 10 minutes to remove calculus and soft tissue debris, and then, the root was debrided on the external surface with a periodontal scaler (Hu-Friedy Mfg. Co., Chicago, Illinois, US).

Standard oval access cavities were prepared on the occlusal surface of each tooth using a carbide bur size #3 (Dentsply Maillefer, Ballaigues, Switzerland) and a high-speedcontra-angle handpiece. Each tooth's working length (WL) was established by entering a K-file size 10 (Mani Inc., Tochigi, Japan) into the root canal until its tip was just visible from the apex, then subtracting 1 mm from that length. A size #15 K-file was placed into each canal to guarantee consistency and avoid teeth with large canals; canals where the file advanced freely to the apical foramen were excluded.

PTU (Dentsply Maillefer, Ballaigues, Switzerland) was utilized for canal instrumentation up to F3 (#30, 0.09). Irrigation was carried out with 3 mL of 5.25% sodium hypochlorite (Clorox Co., 10th of Ramadan, Egypt) following each instrument and finally followed by 3 mL of 17% EDTA for 3 min. The canals were then flushed with 5 mL of 5.25% sodium hypochlorite before being irrigated with 5 mL of distilled water.

As soon as the instrumentation was carried out, the teeth were randomly divided into two groups, in each group *n* = 40 (GI and GII). In GI, a manual #15 K-File was inserted all the way to the apical foramen to make sure of the canal patency. In GII, the same steps were performed but without exceeding 1 mm of the WL.

All canals were obturated with F3 gutta percha cones (Dentsply Maillefer, Ballaigues, Switzerland) and Endosequence BC sealer (Brasseler USA, Savannah, Georgia, USA). To ensure a complete sealer setting, the teeth were placed in an incubator at 37°C with 100% humidity for 7 weeks after filling the access cavities with zinc oxide temporary restoration (Cavit, 3M ESPE).

### 2.1. Preliminary Eppendorf Tube Weight

**Figure 1:**
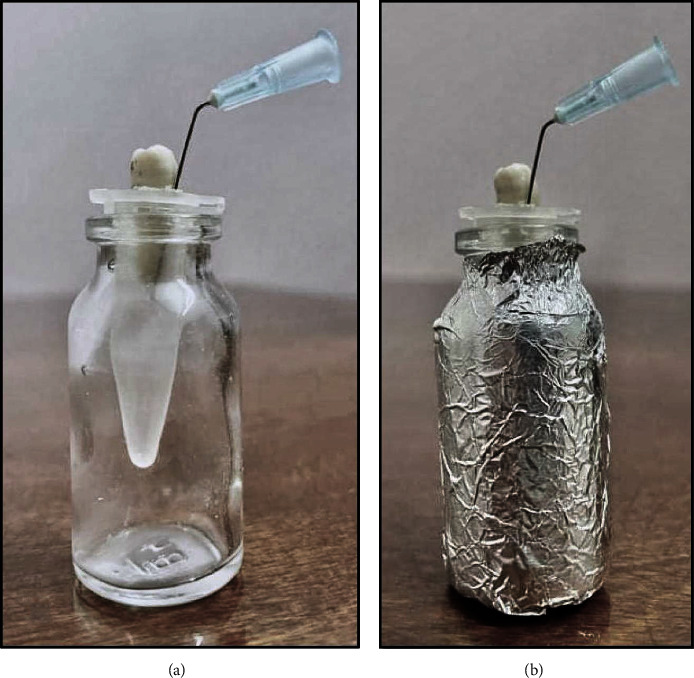
The experimental model used to evaluate debris extrusion (a, b) samples before and after the aluminum foil application.

The Myers and Montgomery method has been adopted in this study to assess debris extrusion during the retreatment procedure [39]. For each tooth, an Eppendorf tube (Eppendorf AG, Hamburg, Germany) was numbered, and a hole in the tube cap was made with a hot instrument. An analytical balance (AUW220D; Shimadzu Analytical Balance, Tokyo, Japan) was used to weigh each Eppendorf tube with a precision of 10^−5^ g. Each Eppendorf tube was weighed three times, and the average of the three weights was used as the tube's preliminary weight. Following that, each root was embedded into the tube cap and sealed on the lateral side with cyanoacrylate to prevent accidental leakage of irrigating solutions during retreatment. For stability and to avoid contamination, each Eppendorf tube was placed in a glass vial. Aluminum foil was then applied to the surface to make it completely opaque, preventing any operator bias. In addition, the tube cap was pierced with a 27-gauge needle to achieve pressure equality, as shown in Figure 1.

### 2.2. Retreatment Protocols and Subgroups

**Figure 2:**
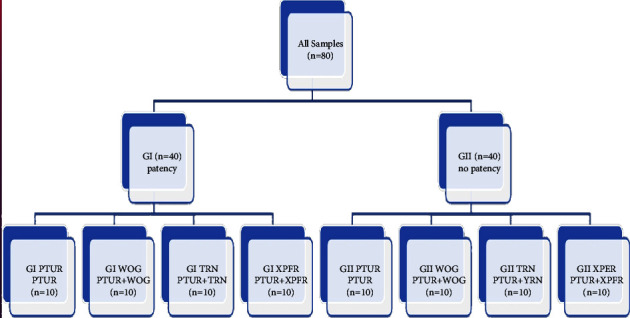
Samples classification.

The two groups of teeth were further subdivided into 8 subgroups (each subgroup *n* = 10): GI PTUR, GI WOG, GI TRN, GI XPFR, GII PTUR, GII WOG, GII TRN, and GII XPFR according to the presence or absence of apical patency (GI and GII) and according to the file system that was adopted to eliminate the previous filling as shown in Figure 2.

#### 2.2.1. GI PTUR and GII PTUR (Control Groups)

PTUR was used to eliminate root fillings from these subgroups (Dentsply Maillefer, Ballaigues, Switzerland) using D1, D2, and D3 files in a pecking motion until reaching WL with D3. All the files were used up to the point where there were no longer observable traces.

#### 2.2.2. GI WOG and WOG GII Groups

Root canal fillings were first removed with PTUR, as was performed with the control groups, and then a WOG medium file (Dentsply, Maillefer, Switzerland) with a 35-mm tip and a 0.06-mm taper was used in reciprocation mode.

#### 2.2.3. GI TRN and GII TRN Groups

PTUR was used first to remove the root canal fillings, as described previously for the control groups, and then TRN medium (Dentsply, Sirona, Ballaigues, Switzerland) size #36 with a 3% taper was operated at 500 rpm with a torque setting of 1.5 Ncm.

#### 2.2.4. GI XPFR and GII XPFR Groups

At first, PTUR was used to remove the root canal fillings, and then XPFR instrument (FKG Dentaire SA, La Chaux-de-Fonds, Switzerland) with tip size 30 and 0 taper was operated at 800 rpm and 1 Ncm torque.

**Figure 3:**
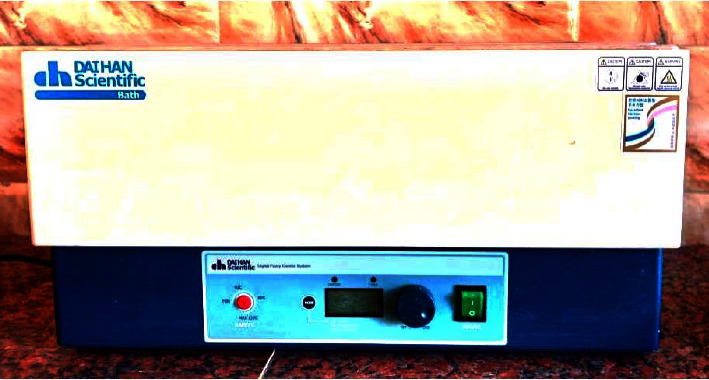
Digital water bath to mimic mouth temperature.

Prior to using the instrument, the plastic tube containing it was cooled with ethyl chloride spray (Walter Ritter GmbH Co. Pharmaceuticals, Germany) to keep it straight while measuring the WL. It was inserted without rotation into the root canal and then activated; the instrument was pressed against the canal walls in three cycles of in-and-out motions up to the WL. For this procedure, the temperature was kept at 37°C inside a digital precise water bath with a digital fuzzy control system (DAIHAN Scientific Co., Ltd. Gangnam-do, Korea) to mimic the body temperature and create ideal conditions for the phase transformation of XPFR instruments Figure 3.

All file systems were operated with the X-Smart Plus endodontic motor (Dentsply Maillefer, Ballaigues, Switzerland) in a rotational or reciprocating motion following the manufacturer's instructions. Irrigation was performed following each instrument with 3 mL of distilled water delivered via a 30-gauge side vented needle to minimize the irrigant extrusion apically (NaviTip, Ultradent, South Jordan, UT, USA). Teeth in GI had their apical patency restored after eliminating the previous canal filling material, as evidenced by the tip of a manual #15 K-File becoming visible in the apical foramen.

### 2.3. The Final Eppendorf Tube Weight

The apical portion of the tooth was washed with 2 mL of distilled water in each tube after the filling material was entirely removed to collect the apically extruded debris that adhered to the external root surface. A preweighed Eppendorf tube was utilized to gather apically extruded debris after the elimination of the canal filling material. After that, the tubes were stored at 70°C for 5 days to allow the irrigation to evaporate. For each subgroup, the weight of dry extruded debris was calculated by subtracting the weight of preretreatment from the weight of postretreatment Eppendorf tubes (the average of three consecutive weights was considered). The quantity of extruded debris from the experimental groups (WOG, TRN, and XPFR) was compared to that of the corresponding control group with the presence or absence of apical patency. One endodontist oversaw the preparation of all samples to reduce differences and get rid of any possible biases.

### 2.4. Statistical Analysis

The normal distribution of data was verified using Levene's Test. The mean quantity of apically extruded debris following different removal techniques was analyzed using the two-way ANOVA followed by post hoc Tukey test for multiple comparisons. Differences between groups were considered statistically significant when *P* ≤ 0.05. Chi-square analysis was conducted to assess the debris extrusion percentage of each supplementary file system in relation to the total quantity of extrusion. All statistical analyses were conducted using Minitab 20 (Minitab Inc., State College, Pennsylvania, USA).

## 3. Results

**Figure 4:**
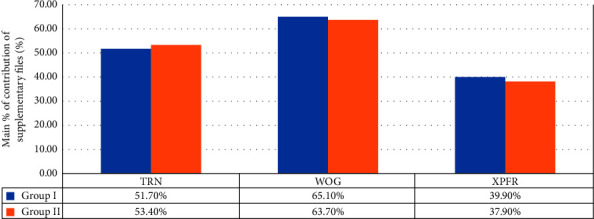
The bar chart showing each supplementary file system's mean % of apically extruded debris from the total extruded debris.

Table 1 summarizes the mean values and standard deviations (SD) for each group's apically extruded debris (gm).  Table 2 depicts the actual quantity of debris extruded by the supplementary retreatment files (WOG, TRN, and XPFR) after subtracting the quantity of apically extruded debris (gm) from the respective control group (GI PTUR and GII PTUR).  Table 3 and Figure 4 display the mean percentage of each supplementary file system's contribution to the total extruded debris.  Table 4 illustrates the results of a two-way ANOVA for the interaction of apical patency on the mean value of debris extrusion following retreatment with the control and supplementary retreatment files.

The results of the twofold retreatment protocol revealed that all supplementary retreatment files extruded debris to varying degrees. When comparing different methods of filling-material removal regardless of apical patency, PTUR + WOG extruded significantly more debris (0.00584 ± 0.00043), followed by PTUR + TRN (0.00438 ± 0.00035) and PTUR + XPFR (0.0034 ± 0.00027) (*P*  <  0.05).

In GI and GII regarding apical patency, no statistically significant difference was found, regardless of how the filling material was removed (GI = 0.00399 ± 0.00146, GII = 0.00385 ± 0.00139). When comparing the actual quantity of debris extruded by the supplementary retreatment files, XPFR groups were associated with the least quantity of debris extrusion (0.00134 ± 0.00031) among all experimental groups. In contrast, WOG groups demonstrated the most quantity of apically extruded debris (0.00378 ± 0.00044). Furthermore, when used as supplementary retreatment files following PTUR, XPFR was associated with the lowest increase in debris extrusion (39.9% ± 4.07 in GI and 37.9% ± 7.64 in GII), while WOG was associated with the highest increase (65.1% ± 2.51 in GI and 63.7% ± 4.81 in GII).

## 4. Discussion

The current study assessed the impact of restoring apical patency or not during the root canal retreatment on the quantitative amount of apically extruded debris following the usage of three contemporary supplementary file systems (WOG, TRN, and XPFR) after PTUR files. It was recommended to re-establish apical patency in endodontically retreated teeth because failed root canal treatments were frequently accompanied by peri-radicular periodontitis or abscess. In such circumstances, accessing the peri-radicular area for microbial removal is crucial to treatment success [14]. The filling material can accumulate at the apical foramen, and the instrument's movement while cleaning can cause more debris to extrude or enhance apical transportation [21].

Extruded debris from using PTUR files that removed most of the primary filling material was used as a control in this study. After using PTUR files, supplemental files were necessary to refine the apical preparation because the diameter of the retreatment D3 file is equal to size #20, precluding acceptable cleaning of the apical portion. Cleaning the root canal with supplemental files helps remove any remaining fillings that may cause debris to be extruded apically, along with previous studies that have shown that the PTUR files alone were insufficient to remove root fillings from the canals; therefore, twofold retreatments were devised [40, 41]. Therefore, in the current study, debris extrusion by supplementary files was compared to that of the control group both in the presence and absence of apical patency.

To eliminate the potential confounding variables, mandibular premolars with a single canal having a degree of curvature less than 20° were chosen, and canal instrumentation was performed using the PTU up to F3 to standardize the samples.

According to the present study results, regaining apical patency did not influence the quantity of extruded debris, irrespective of the filling material elimination methodology, in accordance with Deonizio et al. who proved that apical patency did not affect the volume of material extruded while utilizing the ProTaper rotary system [21]. But these results disagreed with Tinaz et al. who concluded that the manual preparation approaches resulted in more significant extruded debris when applying apical patency [20]. Moreover, multiple investigations revealed a link between apical patency and the extrusion of contaminated debris and irritated periapical tissues [18, 19].

Regardless of the twofold retreatment protocol, all of the instrumentation systems used are accomplished with apically extruded debris. WOG files resulted in a statistically significant increase in apical debris extrusion, followed by TRN, and the smallest quantity produced by XPFR compared to PTUR. Therefore, the hypothesis was rejected. These findings aligned with those of Yilmaz and Ozyurek and Soda et al. since apical extrusion of debris was unavoidable [7, 8].

The control group results were consistent with those of Deonizio et al. and Silva et al. ,which indicated the inadequacy of PTUR files for completely removing root canal fillings and suggested the use of supplemental files [21, 40, 41]. This can be explained as the PTUR system has a convex triangular cross-section with three machined cutting edges, resulting in decreased cutting efficiency and a smaller dentin chip space, which unintentionally acts as a piston and forces debris apically during the instrumentation. Additionally, a D3 file with a 0.2 mm tip diameter and a 7% taper may cause debris to extrude apically [42].

The quantity of apically extruded debris was evaluated while using XPFR instruments during the retreatment procedure following the use of PTUR in the present study due to their remarkable ability to eliminate the previous root canal filling material. The XPFR file system manufacturer claims that these instruments can be used as a universal complementary stage following the canal preparation with any rotary or reciprocating file system. It could also be used as a final step after removing filling material with any file system with a diameter of # 30 or greater [29].

In the present study, the instrument design significantly influences the quantity of debris extruded through the apex. XPFR had significantly less debris extrusion than the other supplementary retreatment groups and the control groups. A possible explanation for this, which was also reported by the manufacturer, was the shape memory principle of MaxWire alloy and its unique triangular cross-section with an off-centered design, which removes debris while preserving the dentine. Additionally, the spoon-like shape at its active tip that expands and contracts to adapt to the canal shape, providing a reach of 6 mm in diameter, or 100-fold greater than a standard instrument of the same size [22, 29, 30].

The XPFR results were consistent with Türker and Kaşıkçı, which showed that using an XPFR file to improve retreatment had no significant influence on the quantity of extruded debris after using a Reciproc file system [31], and Kifr et al. evaluated the debris extrusion of an XPF file when used after the ProTaper Next system in the endodontic treatment and found that the final preparation with an XPF file contributed to an additional quantity of debris [32]. However, the current study's findings could not be directly compared with those studies, either because they were used after the Reciproc file system or because they were not used for the retreatment procedure.

According to the findings of this study, when comparing alternative approaches to filling material removal regardless of apical patency, TRN groups extruded significantly less debris apically than WOG groups did. This finding can be attributed to the heat-treatment and unique geometry of TRN with regressive tapers and an off-centered parallelogram cross-section, which might result in less engagement between the file and dentin, conserving the radicular dentin, thus providing more space for coronal debris extrusion, which finally reduces apical compaction of debris within the canal [21, 33]. Moreover, the kinematics of the file movement may influence the quantity of apical debris extrusion, as more debris was extruded with reciprocating motion than with rotary motion [9].

In addition, WOG medium files also exhibit a larger taper of 0.06 at the apical 3 mm, resulting in the more aggressive preparation of the root canals, which might explain the increased quantity of debris formation and hence its apical extrusion. These results are supported by the findings of Borges et al. and Silva et al. who reported that the larger tapers at the instruments' tips result in an increased quantity of apically extruded debris [43, 44]. And Rabea et al. found more significant debris extrusion produced by the WOG than the PTN system and attributed it to differences in movement kinematics, instrument design, and the number of files between systems [28]. While WOG results contradicted the findings of Delai et al. regarding minimal filling extrusion during the endodontic retreatment, and Azim et al. stated the outcomes of least debris extruded, attributing this to its design, which may not provide adequate space for debris to be displaced, thereby impairing its cutting efficiency [26, 27].

There was a scarcity of data for assessing extruded debris apically during the root canal filling material elimination, using the TRN system as a supplementary approach. Therefore, it was not possible to make direct comparisons. The results of TRN system did not outperform the control groups or the XPFR system in this study. These findings corroborated those of Al Omari et al., who concluded that the TRN system did not surpass the results of other experimental groups. These could be related to the kinematic motion employed and the type of teeth chosen [35]. On the contrary, the present study was not consistent with the findings of Mustafa et al., who found minimal debris extrusion of the TRN system in curved root canals in comparison to other studied groups, and Predin Djuric et al. reported minimal debris apically when TRN was used with its glide path in a clockwise reciprocation motion [36, 37].

In the current study, the main limitation was the absence of simulated back pressure from the periapical tissue so apical extrusion was not limited. Therefore, the results may differ if applied in a clinical situation. Also, the results of this study cannot be generalised to multiple rooted teeth, curved canals, open apices, and incomplete root development cases. Still, it can measure how much debris was extruded from different file systems and help clinicians choose an instrument for the root canal retreatment.

## 5. Conclusions

In conclusion, the apical patency did not affect the quantity of apically extruded debris associated with the studied supplementary single-file systems for eliminating the primary filling material in the current study. In addition, all tested supplementary retreatment files were accompanied by the extrusion of debris apically. XPFR produced the least quantity of extruded debris. Further study is needed to assess the results of this study in a clinical situation.

## Figures and Tables

**Table 1:** The mean ± SD values of apically extruded debris of studied groups (gm).

Groups	PT (control)	(PT + TRN)	(PT + WOG)	(PT + XPFR)
Group I	0.00209 ± 0.00012^A^	0.00438 ± 0.00039^B^	0.00601 ± 0.00031^C^	0.00349 ± 0.00014^D^
Group II	0.00204 **±** 0.00012^A^	0.00439 **±** 0.00031^B^	0.00567 **±** 0.00047^C^	0.00331 **±** 0.00035^D^

The mean values with different superscript letters are statistically different.

**Table 2:** The mean ± SD values of debris extruded by each supplementary retreatment files (gm).

Groups	TRN	WOG	XPFR
Group I	0.00228 **±** 0.00048^b^	0.00391 **±** 0.0003^c^	0.00139 ± 0.00017^d^
Group II	0.00235 **±** 0.00036^b^	0.00363 **±** 0.00053^c^	0.00127 ± 0.0004^d^

The mean values with different superscript letters are statistically different.

**Table 3:** The mean% ± SD values of the contribution of each supplementary file system from the total extruded debris.

Groups	TRN	WOG	XPFR
Group I	51.7% ± 6.39	65.1% ± 2.51	39.9% ± 4.07
Group II	53.4% ± 4.79	63.7% ± 4.81	37.9% ± 7.64

**Table 4:** Two-way ANOVA analysis for the interaction of apical patency on the mean value of apical extrusion after the retreatment with supplementary contemporary retreatment files.

Source	Type III sum of squares	*df*	Mean square	*F*	Sig.
Corrected model	0.000^a^	7	2.185*E* − 5	236.782	0.000
Intercept	0.001	1	0.001	13330.952	0.000
Patency	4.118*E* − 7	1	4.118*E* − 7	4.462	0.038
Apical extrusion	0.000	3	5.073*E* − 5	549.685	0.000
Patency *∗* apical extrusion	3.656*E* − 7	3	1.219*E* − 7	1.321	0.274
Error	6.645*E* − 6	72	9.230*E* − 8		
Total	0.001	80			
Corrected total	0.000	79			

^a^ *R* squared = 0.958 (adjusted *R* squared = 0.954).

## Data Availability

The data used to support the findings of this study are included within the article and are available from the corresponding author upon reasonable request.

## Additional Points

*Clinical Relevance.* The best procedure for removing previous root canal filling material, either with or without re-establishing apical patency, should be determined to minimize or eliminate apically extruded debris. Thus, better postoperative outcomes and greater levels of patient satisfaction are possible.
